# Supplementary material for: Chaperone-Mediated Autophagy Markers LAMP2A and HSPA8 in Advanced Non-Small Cell Lung Cancer after Neoadjuvant Therapy
Source: Cells. 2021 Oct 13;10(10):2731. doi: 10.3390/cells10102731 (PMC8534862; doi:10.3390/cells10102731)
Supplement: Supplementary file 1 [file cells-10-02731-s001.zip › cells-1369290-supplementary/Supplementary Figures S1-S3/Figure S2.pdf]

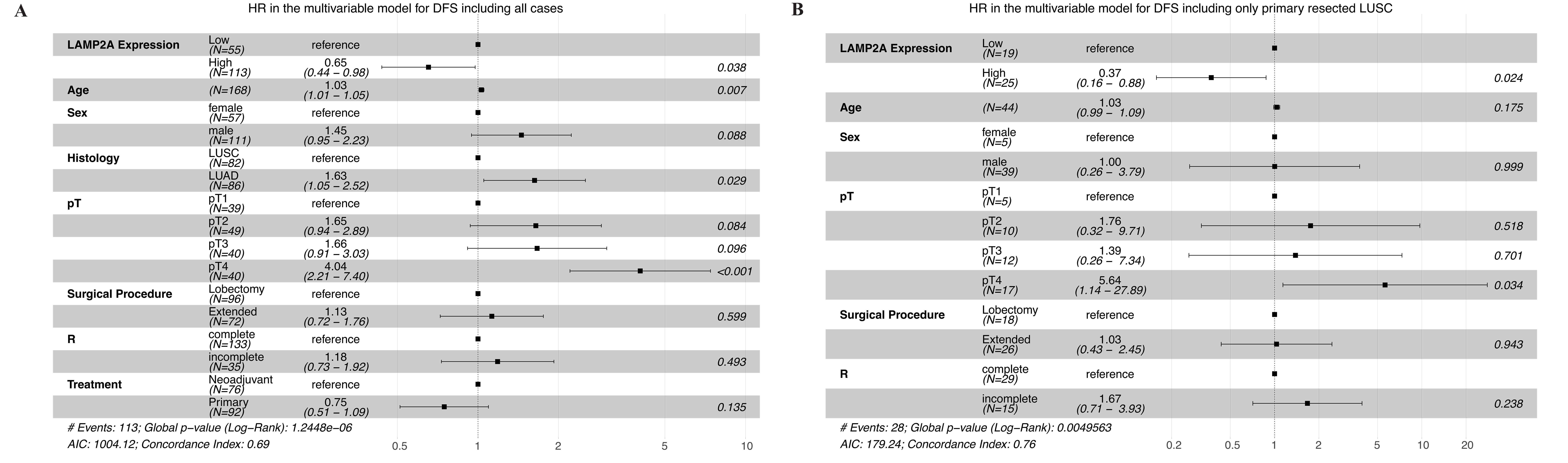

A
HR in the multivariable model for DFS including all cases
LAMP2A Expression
Low (N=55) reference
High (N=113) 0.65 (0.44 – 0.98) 0.038
Age (N=168) 1.03 (1.01 – 1.05) 0.007
Sex
female (N=57) reference
male (N=111) 1.45 (0.95 – 2.23) 0.088
Histology
LUSC (N=82) reference
LUAD (N=86) 1.63 (1.05 – 2.52) 0.029
pT
pT1 (N=39) reference
pT2 (N=49) 1.65 (0.94 – 2.89) 0.084
pT3 (N=40) 1.66 (0.91 – 3.03) 0.096
pT4 (N=40) 4.04 (2.21 – 7.40) <0.001
Surgical Procedure
Lobectomy (N=96) reference
Extended (N=72) 1.13 (0.72 – 1.76) 0.599
R
complete (N=133) reference
incomplete (N=35) 1.18 (0.73 – 1.92) 0.493
Treatment
Neoadjuvant (N=76) reference
Primary (N=92) 0.75 (0.51 – 1.09) 0.135
Events: 113; Global p-value (Log-Rank): 1.2448e-06
AIC: 1004.12; Concordance Index: 0.69
0.5
1
2
5
10
B
HR in the multivariable model for DFS including only primary resected LUSC
LAMP2A Expression
Low (N=19) reference
High (N=25) 0.37 (0.16 – 0.88) 0.024
Age (N=44) 1.03 (0.99 – 1.09) 0.175
Sex
female (N=5) reference
male (N=39) 1.00 (0.26 – 3.79) 0.999
pT
pT1 (N=5) reference
pT2 (N=10) 1.76 (0.32 – 9.71) 0.518
pT3 (N=12) 1.39 (0.26 – 7.34) 0.701
pT4 (N=17) 5.64 (1.14 – 27.89) 0.034
Surgical Procedure
Lobectomy (N=18) reference
Extended (N=26) 1.03 (0.43 – 2.45) 0.943
R
complete (N=29) reference
incomplete (N=15) 1.67 (0.71 – 3.93) 0.238
Events: 28; Global p-value (Log-Rank): 0.0049563
AIC: 179.24; Concordance Index: 0.76
0.2
0.5
1
2
5
10
20
