# Supplementary figures and images for: Chaperone-Mediated Autophagy Markers LAMP2A and HSPA8 in Advanced Non-Small Cell Lung Cancer after Neoadjuvant Therapy
Source: Cells. 2021 Oct 13;10(10):2731. doi: 10.3390/cells10102731 (PMC8534862; doi:10.3390/cells10102731)

**A**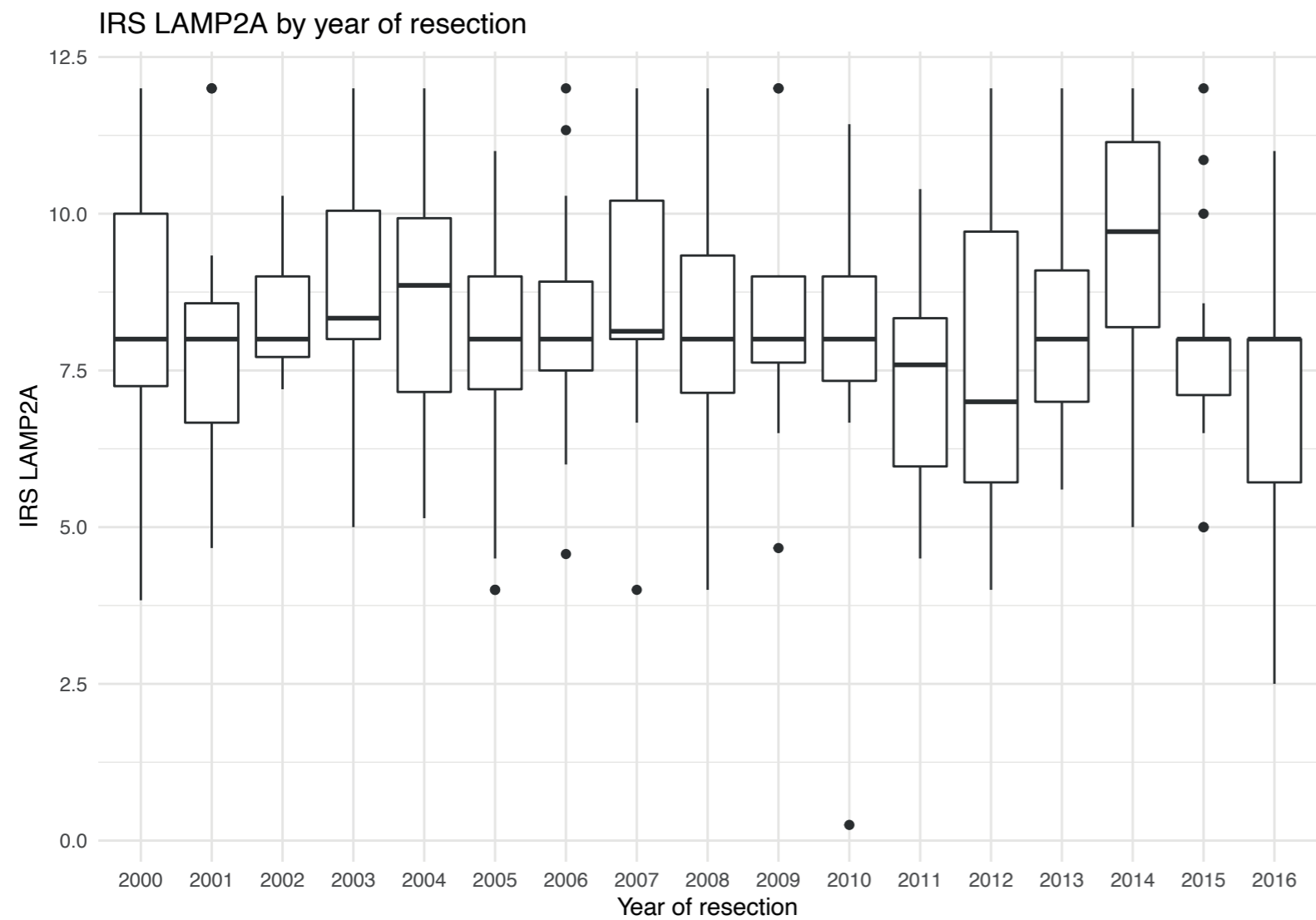**B**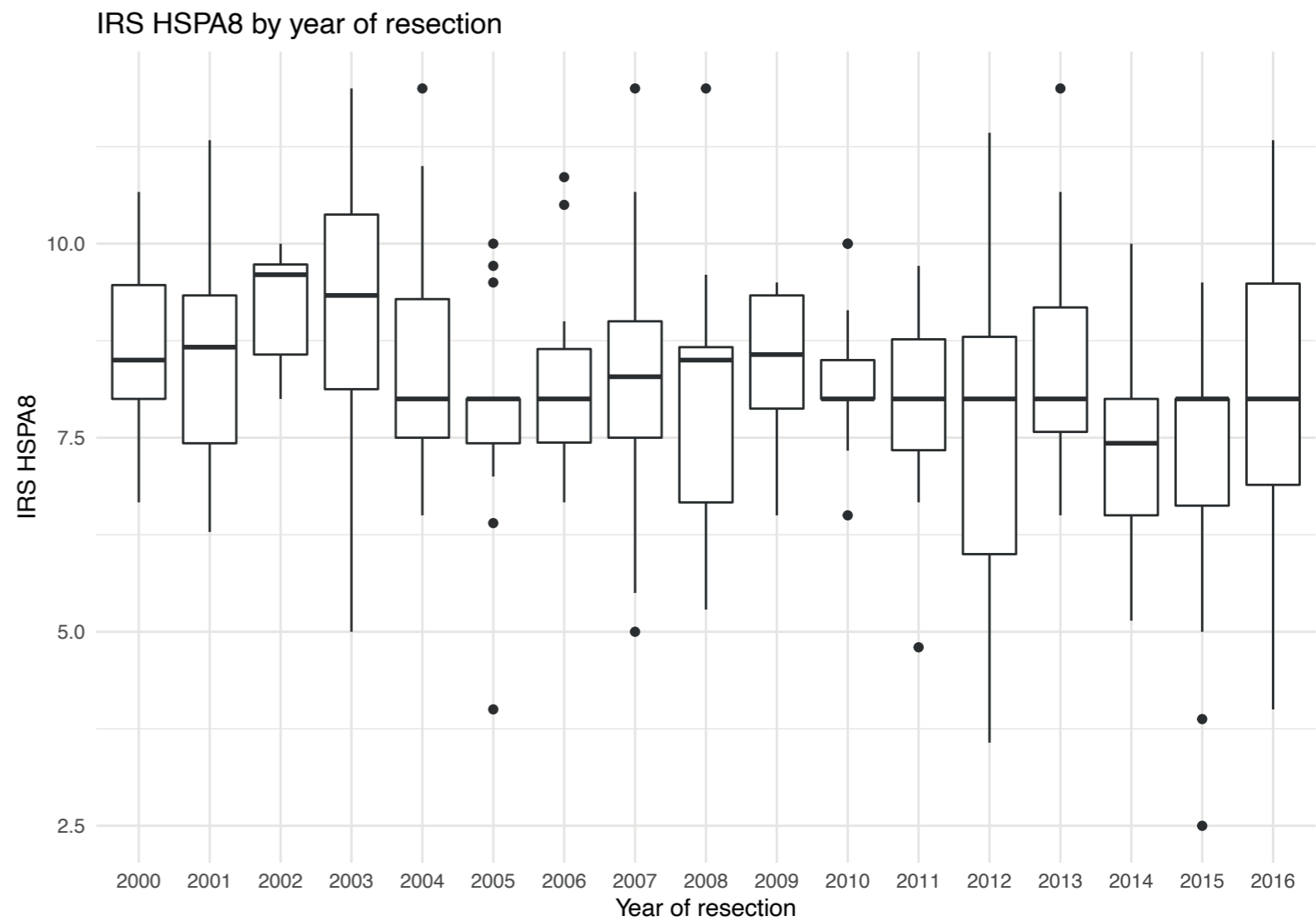

Supplement: Supplementary file 1 [file cells-10-02731-s001.zip › cells-1369290-supplementary/Supplementary Figures S1-S3/Figure S3.pdf]
